# Changing epidemiology of cirrhosis from 2010 to 2019: results from the Global Burden Disease study 2019

**DOI:** 10.1080/07853890.2023.2252326

**Published:** 2023-08-30

**Authors:** Shiyu Xiao, Wenhui Xie, Yinghui Zhang, Lei Lei, Yan Pan

**Affiliations:** a Department of Gastroenterology, Sichuan Provincial People’s Hospital, Chengdu, China; b Department of Rheumatology, Peking University First Hospital, Beijing, China

**Keywords:** Cirrhosis, epidemiology, global burden of disease

## Abstract

**Background:**

Liver cirrhosis is a significant yet largely preventable and underappreciated cause of global health loss. This study aimed to profile the global and regional burdens of liver cirrhosis between 2010 and 2019 and the contributions of various aetiologies.

**Method:**

Data on the incidence, mortality, and disability-adjusted life years (DALYs) of cirrhosis were obtained from the Global Burden of Disease 2019 study. The burden of cirrhosis was estimated by age, sex, region, aetiology, and socio-demographic index (SDI). The temporal trend was quantified using the annual percentage changes (APC.)

**Results:**

Globally, there were 2.05 million new cases and 1.47 million deaths due to cirrhosis in 2019. From 2010 to 2019, the age-standardized incidence rate (ASIR) for cirrhosis increased slightly from 25.19 to 25.35 worldwide, while the age-standardized death rate (ASDR) and age-standardized DALYs (ASDALYs) decreased from 20.37 to 18.00 and 639.86 to 560.43, respectively. Cirrhosis incidence, mortality and DALYs were consistently higher in males than females. Stratification according to the socio-demographic index (SDI) revealed that low SDI countries had the highest ASDR and ASDALYs in 2019, while middle SDI countries had the highest ASIR. Regarding the aetiology of cirrhosis, hepatitis C accounted for the largest proportion of cirrhosis-related incidence (26.9%), death (26.8%) and DALYs (26.3%); however, non-alcoholic fatty liver disease (NAFLD) exhibited a rapidly growing cause of incident cirrhosis (+26.7%), cirrhosis-related death (+25.1%), and DALYs (+21.0%) worldwide during this period. The ASIR for NAFLD also significantly increased with APC 1.080 over the study period.

**Conclusions:**

Albeit the global burden of cirrhosis incidence increased from 2010 to 2019, cirrhosis-associated deaths and DALYs declined significantly. Notably, NAFLD exhibited the most significant increase as a contributor to cirrhosis worldwide.

## KEY MESSAGE

- Global burden of cirrhosis incidence increased from 2010 to 2019.
- Cirrhosis-associated death and DALYs declined significantly during this period.
- Non-alcoholic fatty liver disease (NAFLD) exhibited the most significant increase as a contributor to cirrhosis worldwide.

## Introduction

Cirrhosis is a significant yet largely preventable disease; however, it has received less public attention than other chronic diseases such as chronic kidney disease, diabetes, and congestive heart disease [1]. Cirrhosis typically develops after a long period of liver inflammation, replacing healthy liver parenchyma with fibrotic tissues and regenerative nodules [2]. The disease evolves from an asymptomatic phase (compensated cirrhosis) to a symptomatic phase (decompensated cirrhosis), the complications of which often lead to hospitalization, impaired quality of life, and high mortality [3]. Globally, cirrhosis ranked among the top 20 causes of global health loss, accounting for 1 million deaths in 2010, 1.6% of disability-adjusted life years (DALYs), and 2.1% of the total years of life lost in the global mortality burden [4,5]. Thus, it is crucial to fully understand the burden of cirrhosis and develop policies and strategies to deal with this chronic disease more effectively.

Over the last two decades, numerous socioeconomic and clinical changes have occurred worldwide, which might impact the aetiological profiles of cirrhosis. Notably, highly effective antiviral treatments for hepatitis B virus (HBV) and hepatitis C virus (HCV) have decreased the global burden of viral hepatitis [6]. On the contrary, the increasing prevalence of non-alcoholic fatty liver disease (NAFLD) parallels the obesity and type 2 diabetes epidemic worldwide, especially in Western countries [7,8]. In this context, these conditions may cause a shift in the global epidemiology of cirrhosis.

Previous studies based on the Global Burden of Disease (GBD) 2017 estimated the cirrhosis burden in terms of its prevalence, death, and DALYs [9–11]. However, limited data reported the burden of incident cirrhosis. Recently, studies have also reported the epidemiology of cirrhosis caused by specific aetiologies, such as HBV infection and alcohol use [6,12,13]. Nevertheless, there is a lack of updated and in-depth analysis of the temporal trends in liver cirrhosis incidence, mortality, and DALYs and the contributions of various aetiologies at the global, regional, and national levels. Therefore, we used the data from the latest GBD study 2019 to analyze the global epidemiology of liver cirrhosis in 204 countries and territories, considering their associations with socioeconomic status. This systematic assessment aims to offer a more comprehensive outlook, facilitating the development of global and regional health policies geared towards reducing the burden of this chronic disease.

## Methods

### Data source

This study was performed using public data from the GBD 2019 study, which provides systematic assessments of the burden of 369 diseases and injuries and 87 risk factors from 204 countries and territories [14]. For this study, we obtained the annual absolute count and age-standardized rates (with 95% uncertainty interval [UI]) of liver cirrhosis-related incidence, death, and DALYs from 2010 to 2019 by sex, age, region, and country from the online tool, the Global Health Data Exchange query tool (https://vizhub.healthdata.org/gbd-results/). The estimated population data independently produced by the GBD study was used as the reference for calculating the age-standardized rate [15].

### Estimation methods in GBD study

The detailed methods for gathering, processing, and producing these data have been described extensively in the GBD 2019 study [14]. The methodology of cirrhosis burden estimation was described comprehensively in the GBD 2017 study [9]. The aetiologies of cirrhosis were categorized into different groups in the GBD 2019 study, including alcohol, HBV, HCV, NAFLD, and other chronic liver diseases. The category labelled “other chronic liver diseases” encompasses conditions such as autoimmune hepatitis, toxic liver diseases, other inflammatory liver diseases, chronic hepatitis not specified, and other diseases of the liver.

To assess the association between development level and cirrhosis burden, the socio-demographic index (SDI) (ranging from 0 [worst] to 1.0 [best]) that integrates the total fertility rate, average educational attainment among the population over age 15, and measures of income per capita were used to group countries with similar development status (including low, low-middle, middle, high-middle, and high SDI). In the present study, SDI data were directly obtained from the GBD dataset (https://ghdx.healthdata.org/record/ihme-data/gbd-2019-socio-demographic-index-sdi-1950-2019).

### Statistical analysis

Data were analyzed and displayed using R (Version .3.5.3, R Core Team). The changes in the frequency of incidence, death, and DALYs between 2010 and 2019 were calculated as follows: [(values in 2019) − (value in 2010)]/value in 2010. Smoothing spline models were employed to determine the shape of the correlation curve between cirrhosis burden (age-standardized rates) and SDIs.

To quantitatively evaluate the trend of age-standardized rates over a specified period, the estimated annual percentage change (APC) with a 95% confidence interval (CI) in age-standardized rates was calculated using the Joinpoint regression program (Statistical Research and Applications Branch, National Cancer Institute, Bethesda). Briefly, age-standardized rates with standard errors for each year were used to estimate the APC using log transformation. The model was built using the grid search method, and the significance was tested using the Monte Carlo permutation test. Given that the study’s time frame was from 2010 to 2019, the number of Joinpoint was set to two. An APC estimation above 0 indicates a statistically significant increasing age-standardized rate over time, while a value below 0 indicates a statistically significant decreasing age-standardized rate. If the 95% confidence intervals (CIs) of the APC include 0, there is no statistically significant change in the age-standardized rate over the study period.

## Results

### Global, regional and national epidemiology of cirrhosis from 2010 to 2019

#### Incidence

**Figure 1.**
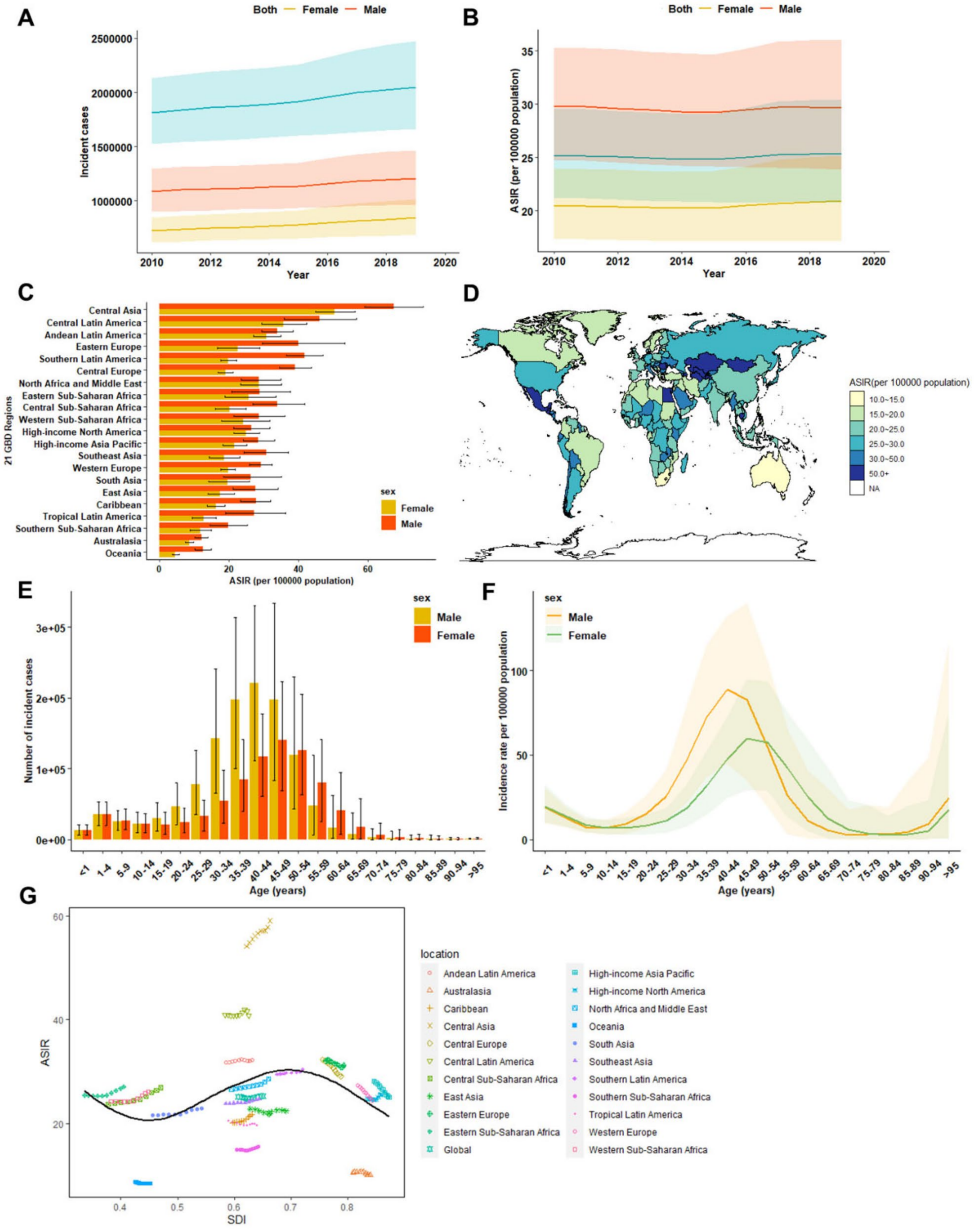
The burden of cirrhosis incidence from 2010 to 2019. A. Temporal trend in the number of new cirrhosis cases at the global level by sex. B. Temporal trend in the age-standardized incidence rate of cirrhosis by sex. C. The age-standardized incidence rate of cirrhosis in 2019 across 21 GBD regions, by sex. D. The age-standardized incidence rate of cirrhosis in 2019, by countries and territories. E. The number of incident cirrhosis by sex in different age groups. F. The age-standardized incidence rate of cirrhosis by sex in different age groups. G. The age-standardized incidence rate of cirrhosis across 21 GBD regions, by socio-demographic index. ASIR age-standardized incidence rate, NA non-available, SDI socio-demographic index.

A total of 2051554 (95% UI 1661430 to 2478127) new cases of cirrhosis were reported in 2019, exhibiting a 12.9% increase compared to 2010 (Figure 1A; Supplementary Table 1). During this period, the APC in the age-standardized incidence rate (ASIR) was 0.104 (95%CI 0.001 to 0.204) at the global level (Supplementary Table 1). At the regional level, Central Asia (59.06 [95% UI 52.30 to 66.01] per 100 000 population) exhibited the highest ASIR of cirrhosis in 2019, while the lowest ASIR was observed in Oceania (8.5 [95% UI 7.05 to 10.00] per 100 000 population) (Figure 1C; Supplementary Table 1). At national level, the countries with the highest ASIR included Republic of Moldova (79.96 [95% UI 71.72 to 86.27] per 100 000 population), Mongolia (79.64 [95% UI 66.44 to 94.18] per 100 000 population) and Egypt (78.71 [95% UI 62.25 to 99.00] per 100 000 population); while the countries with the lowest ASIR were Papua New Guinea (6.07 [95% UI 4.92 to 7.23] per 100 000 population) and Cook Islands (6.07 [95% UI 5.00 to 7.13]) (Figure 1D; Appendix 1). From 2010 to 2019, the largest increase in national ASIR was found in Kazakhstan (APC:3.431[95% CI 3.300 to 3.562]), while the largest decrease was found in Taiwan (Province of China) (APC: −2.706 [95%CI −2.869 to −2.543]) (Appendix 1).

Regarding the age and sex patterns of cirrhosis incidence burden, new cirrhosis cases and ASIR were higher in males (incident cases:1.21 million [95% UI 0.96 to 1.46]; ASIR: 29.67[95% UI 23.86 to 35.98]) than in females (incident cases: 0.85 million [95% UI 0.69 to 1.02]; ASIR: 20.14 [95%UI 17.22 to 25.15]) worldwide in 2019 (Figure 1(A—B)**;** Supplementary Table 2). Similarly, a higher age-standardized rate of cirrhosis incidence was observed in males across almost all GBD regions (Figure 1C). Analysis of age patterns revealed that cirrhosis incidence peaked at 40–44 years in males and 45–49 years in females, with a higher incidence in females than in males after 45 years of age (Figure 1E). The ASIR increased up to 40–44 years for males and 45–49 years for females and decreased until 80-84 years for males before rapidly increasing again (Figure 1F). Additionally, the incidence rate was higher in females less than 4 years and in the 54–79 year age group compared to males (Figure 1F).

After stratification according to the socio-demographic index (SDI), in 2019, the highest frequency of incident cases (*n* = 551689) and the highest ASIR were observed in the middle SDI countries (Supplementary Table 2). Between 2010 and 2019, the greatest increase in the incident cases (+26.7%) occurred in high-middle SDI countries, with the most significant increase in the ASIR (APC:0.78, 95% CI 0.67 to 0.89) observed in low SDI countries, while the greatest reduction in ASIR was observed in high-SDI countries (APC: −0.657 [95% CI −0.737 to −0.576]). For the association of age-standardized rates versus SDI at the regional level, the ASIR of cirrhosis peaked at an SDI of 0.7, due to the high ASIR in the Central Asian region (Figure 1G). Moreover, this region exhibited a sustained increase in the age-standardized incidence of cirrhosis over the study period, which was significantly higher than expected based on the SDI.

#### Death

**Figure 2.**
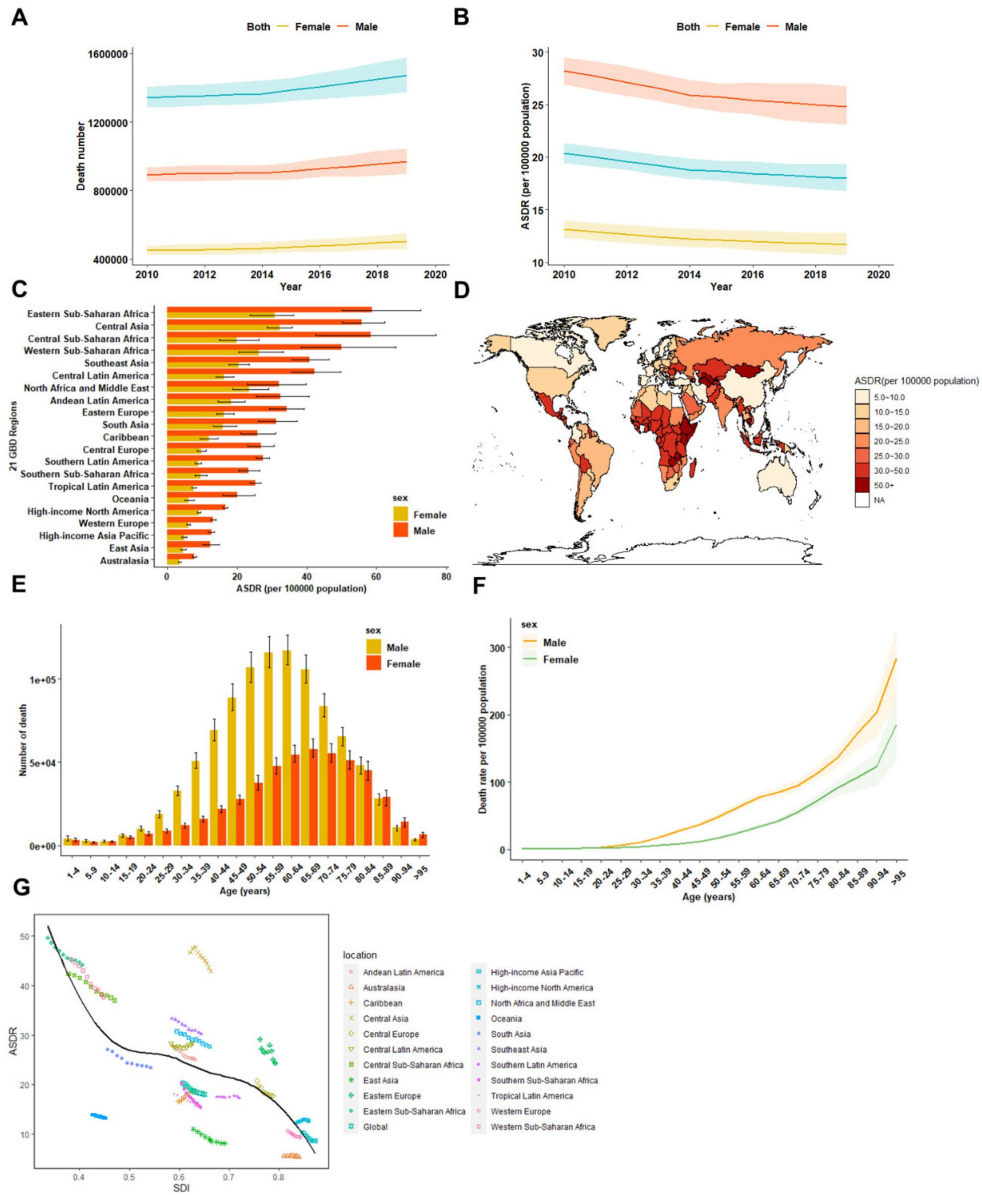
The burden of cirrhosis-related death from **2010 to 2019**. A. Temporal trend in the number of death due to cirrhosis at the global level by sex. **B**. Temporal trend in the age-standardized death rate of cirrhosis by sex. **C**. The age-standardized death rate of cirrhosis in 2019 across 21 GBD regions, by sex. **D**. The age-standardized death rate of cirrhosis in 2019, by countries and territories. **E**. The number of cirrhosis death by sex in different age groups. **F**. The age-standardized death rate of cirrhosis by sex in different age groups. **G**. The age-standardized death rate of cirrhosis across 21 GBD regions, by socio-demographic index. ASDR age-standardized death rate, NA non-available, SDI socio-demographic index.

Globally, cirrhosis caused more than 1.47 million (95% UI 1.37 to 1.58) deaths in 2019, compared with 1.34 million (95%UI 1.29 to 1.41) in 2010 (Figure 2A; Supplementary Table 3). These deaths constituted 2.6% (1472012/56526959) of all deaths worldwide in 2019. The age-standardized death rate (ASDR) at the global level decreased from 20.37 (95%UI 19.45 to 21.36) per 100 000 population in 2010 to 18.00 (95%UI 16.8 to 19.3) per 100 000 populations in 2019, with an APC of −1.392 (95%CI −1.442 to −1.343) worldwide (Figure 2B; Supplementary Table 3). At the regional level, Eastern Sub-Saharan Africa (44.10 [95%UI 38.5-51.9]) ranked first in terms of ASDR from cirrhosis across the GBD regions in 2019 (Figure 2C). The APC in the ASDR from 2010 to 2019 across these GBD regions is presented in Supplementary Table 3, exhibiting a significant decrease in East Asia (APC-3.288 [95%CI −3.483 to −3.093]). Egypt (126.7 [95% UI 87.3 to169.5]) exhibited the highest ASDR of cirrhosis in 2019, while Iceland (3.3 [95% UI 2.9 to 3.7]) and Singapore (3.3 [95% UI 2.9 to 3.6]) exhibited the lowest ASDR (Figure 2D; Appendix 2).

Global death due to cirrhosis was also higher among males (0.97 million [95% UI 0.90 to 1.05]) than females (0.50 million [95% UI 0.46 to 0.55]) (Figure 2A; Supplementary Table 4). Similar to the frequency of death, the mortality rate in males was 2.1-fold higher (males 24.81[95%UI 23.07 to 26.75] *versus* females 11.69 [95% UI 10.68 to 12.81]) than in females (Figure 2B; Supplementary Table 4). Regarding the age patterns in cirrhosis death, the number of deaths peaked in the 60-64 age group for males and 65–69 age group for females (Figure 2E), whereas the ASDR increased steadily with ageing, peaking in the 95 plus age group for both genders in 2019 (Figure 2F).

In terms of development level and its correlation to cirrhosis-related death burden, the highest frequency of deaths (*n* = 395022) was also observed in the middle SDI countries (Supplementary Table 4), while low SDI countries had the highest ASDR in 2019 (Supplementary Table 4). From 2010 to 2019, the greatest increase in death rate (+25.1%) occurred in high-middle SDI countries, whereas ASDR generally decreased at different levels of SDI (Supplementary Table 4). Notably, ASDR was lower at the regional level at higher SDI levels, with the expected rates following a roughly linear decreasing trend **(**Figure 2G**)**. Specifically, from 2010 to 2019, the ASDR decreased or remained steady in most regions, except for the Caribbean and Central Latin America. In these two regions, the observed ASDR increased during the study period, and the rate in Central Latin America increased to a much higher level than expected, based solely on the SDI.

#### DALYs

**Figure 3.**
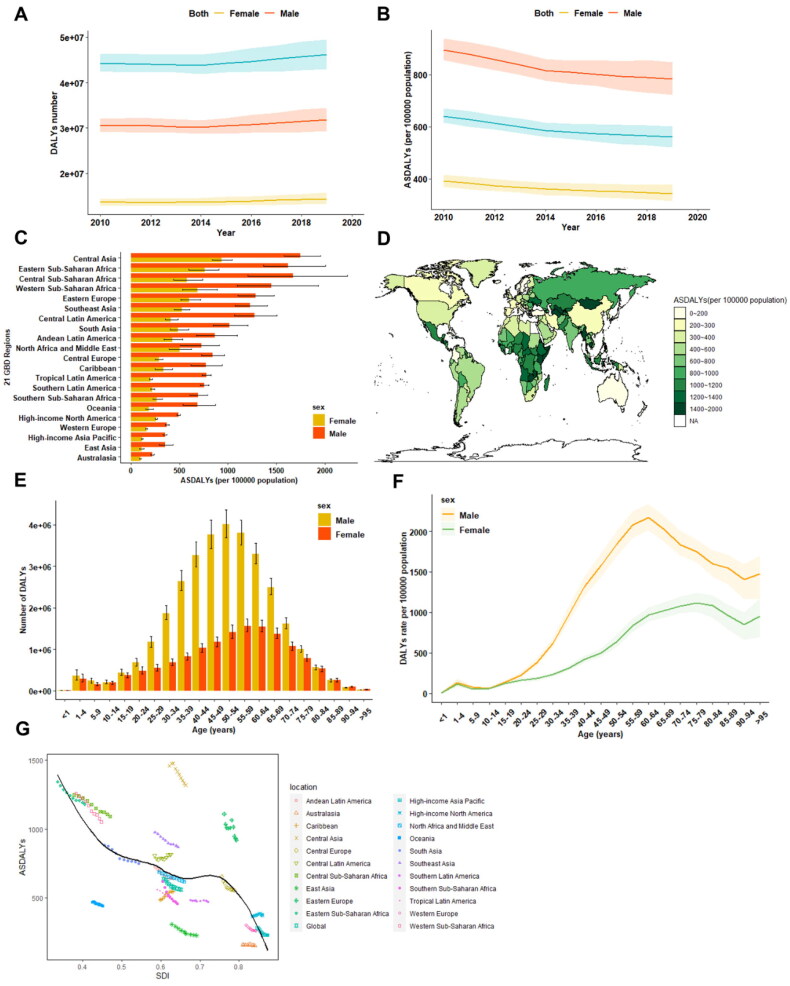
The burden of cirrhosis-associated DALYs from **2010 to 2019**. **A**. Temporal trend in the number of DALYs due to cirrhosis at the global level by sex. **B**. Temporal trend in the age-standardized DALYs rate of cirrhosis by sex. **C**. The age-standardized DALYs rate of cirrhosis in 2019 across 21 GBD regions, by sex. **D**. The age-standardized DALYs rate of cirrhosis in 2019, by countries and territories. **E**. The number of DALYs due to cirrhosis by sex in different age groups. **F**. The age-standardized DALYs rate of cirrhosis by sex in different age groups. **G**. The age-standardized DALYs rate of cirrhosis across 21 GBD regions, by socio-demographic index. ASDALYR age-standardized DALYs rate, NA non-available, SDI socio-demographic index.

Cirrhosis led to a significant increase in DALYs in 2019 (*n* = 46189416, 95% UI 43027109 to 49551292) compared to 2010 (*n* = 44110812, 95% UI 42448751 to 46337063)(Figure 3A; Supplementary Table 5). The age-standardized DALYs (ASDALYs) decreased from 639.86 (95% UI 615.71 to 671.51) in 2010 to 560.43 (95% UI 521.86 to 602.02) per 100 000 populations in 2019 worldwide (APC: −1.498 [95%CI −1.557 to −1.439]) (Figure 3B; Supplementary Table 5). At the regional level, Central Asia (1318.2 [95% UI 1187.0 to 1467.1]) ranked first among 21 GBD regions in terms of ASDALYs (Figure 3C; Supplementary Table 5). In line with the ASDR at the national level, Egypt (2409.7 [95% UI 1570.9 to 3247.4]) was also among the countries and territories with the highest ASDALYs of cirrhosis in 2019, and the lowest ASDALYs of cirrhosis were found in Singapore (81.5 [95% UI 74.7 to 89.0]) (Figure 3D; Appendix 3).

Stratification according to gender revealed that the number of DALYs was also higher in males (31.78 million [95% UI 29.37 to 34.44]) than in females (14.41 million [95% UI 13.16 to 15.76]) (Figure 3A; Supplementary Table 6) in 2019. The DALYs rate was 2.3-fold higher in males (783.31 [95% UI 723.85 to 849.07]) compared to their female counterpart (343.96 [95% UI 313.74 to 376.74]) (Figure 3B; Supplementary Table 6). Regarding the age pattern, the number of DALYs peaked at 50–54 years in males and 55-59 years in females (Figure 3E), whereas the highest age-standardized DALYs rate was found in the 56-59 age group in males and the 70–74 age group in females (Figure 3F).

When assessing the association between DALYs and SDI, it was similar to those of death patterns. The highest frequency of DALYs (1.4 million) in 2019 occurred in the middle SDI countries (Supplementary Table 6), while low SDI countries were associated with the highest ASDALY in 2019 (Supplementary Table 6). (Figure 3G). Between 2010 and 2019, the most significant increase in death rates occurred in high-middle SDI countries (+25.1%), while ASDALYs generally decreased at different levels of SDI (Supplementary Table 6). Notably, the most significant reduction in ASDALYs occurred in high-middle SDI countries (APC: −2.559 [95% CI −3.164 to −2.030]) (Supplementary Table 6).

### Global and regional trends in the aetiology of cirrhosis

**Figure 4.**
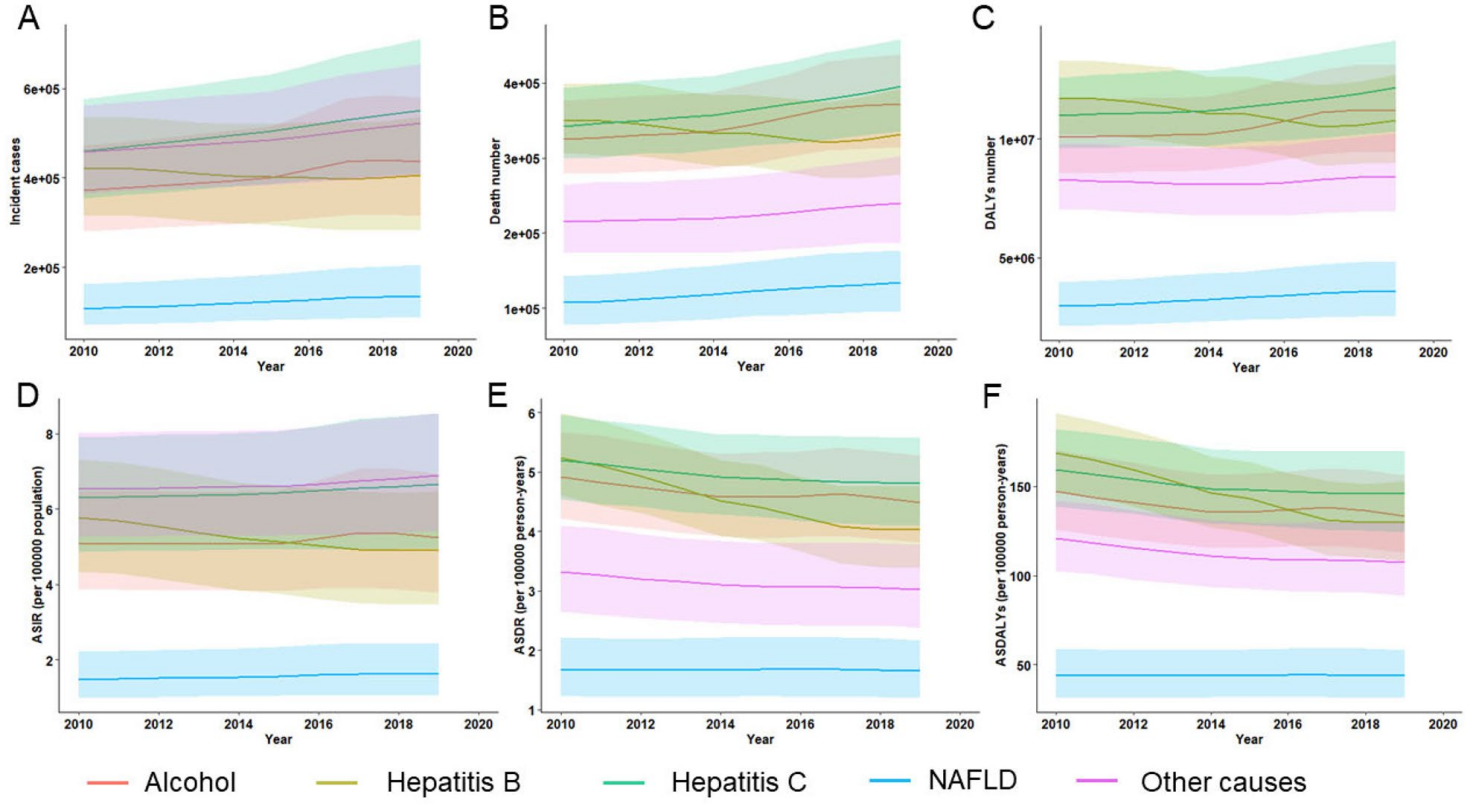
The global trends in the number and age-standardized rates of incidence, death and DALYs of cirrhosis stratified by different causes from **2010 to 2019. A** to **C**. Global trends in the number of incidence (**a**), death (**B**) and DALYs (**C**) of cirrhosis caused by alcohol use, HBV, HCV, NAFLD and other reasons over the study period. **D** to **F**. Global trends in the age-standardized rates of incidence (**D**), death (**E**) and DALYs (**F**) of cirrhosis due to different causes from 2010 to 2019. ASIR age-standardized incidence rate, ASDR age-standardized death rate, ASDALYs age-standardized DALYs rate.

**Figure 5.**
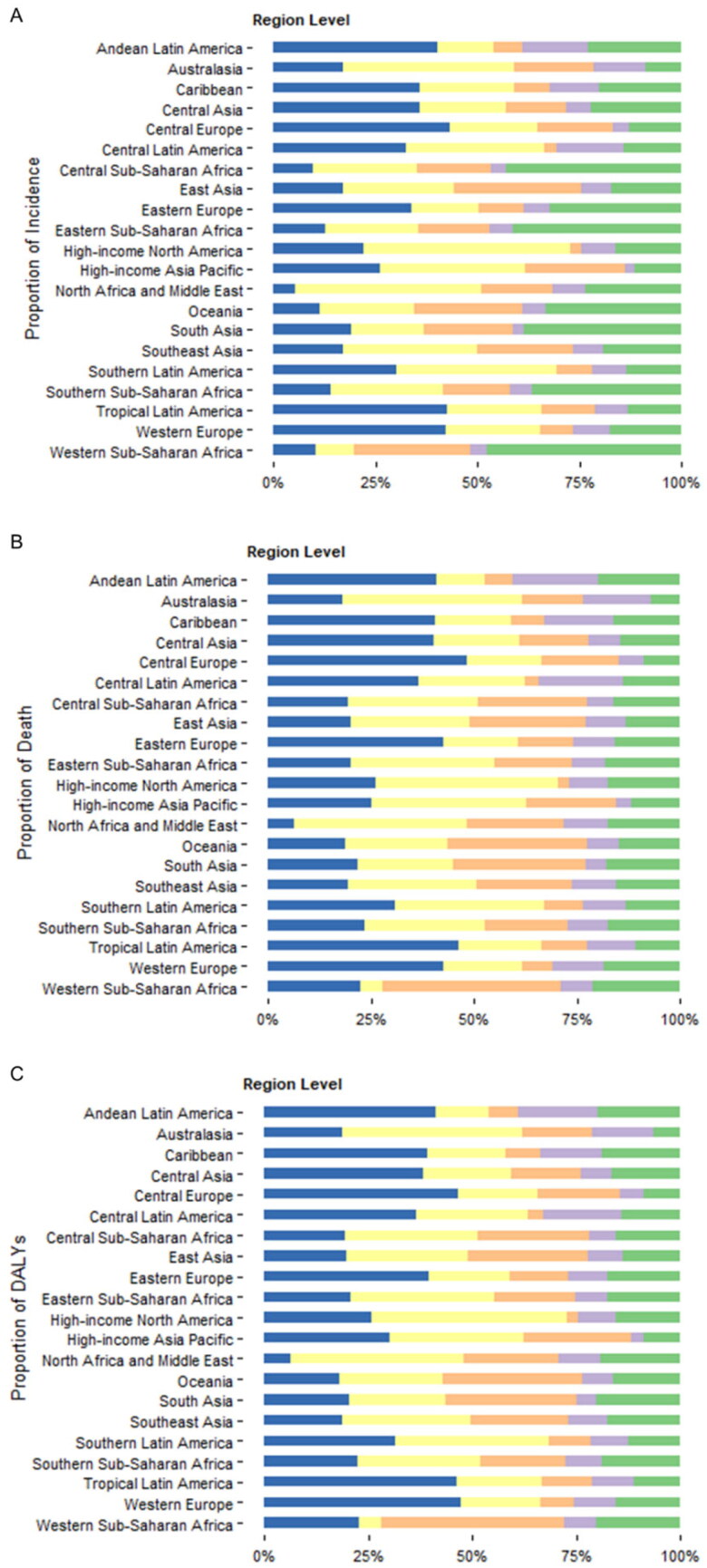
Proportion of incidence (a), death (B) and DALYs (C) due to five causes of cirrhosis at regional levels in **2019.** DALYs disability-adjusted life years.

The global frequencies of incident cases, deaths, and DALYs by five aetiologies (alcohol, HBV, HCV, NAFLD, and other causes combined) of cirrhosis from 2010 to 2019 are presented in Figure 4. For the global incident cases in 2019, HCV (26.9%) accounted for the largest proportion of global liver cirrhosis, followed by alcohol (21.3%), HBV (19.8%), and NAFLD (6.6%); other causes were responsible for 25.4% of incident cases (Supplementary Table 2). In this respect, NAFLD exhibited the most significant increase as a cause of incident cirrhosis (+26.7%) worldwide between 2010 and 2019, while the absolute count of HBV-related cirrhosis decreased (-0.34%) during this period (Supplementary Table 2). For the cause of cirrhosis-related death, HCV was also the main cause of cirrhosis-related death (26.8%) and DALYs (26.3%) in 2019, while NAFLD has become the fast-growing cause of cirrhosis-related death (+25.1%) and DALYs (+21.0%) worldwide (Supplementary Tables 4 & 6). There were wide geographic variations in the contribution of each cause to the incidence of cirrhosis and death in 2019. Across the GBD regions, the incidence of and death from cirrhosis due to alcohol use was highest in Central Europe (incidence 33.8%, death 42.5%) and lowest in North Africa and the Middle East (incidence 5.2%, death 6.4%) (Figure 5(A,B). For virus-related cirrhosis, HCV was the main cause of cirrhosis incidence and associated death in high-income North America, whereas HBV-related cirrhosis death and DALYs were the highest in Western Sub-Saharan Africa, although HBV was not the main cause of cirrhosis incidence in this region (Figure 5(A,B).).

**Figure 6.**
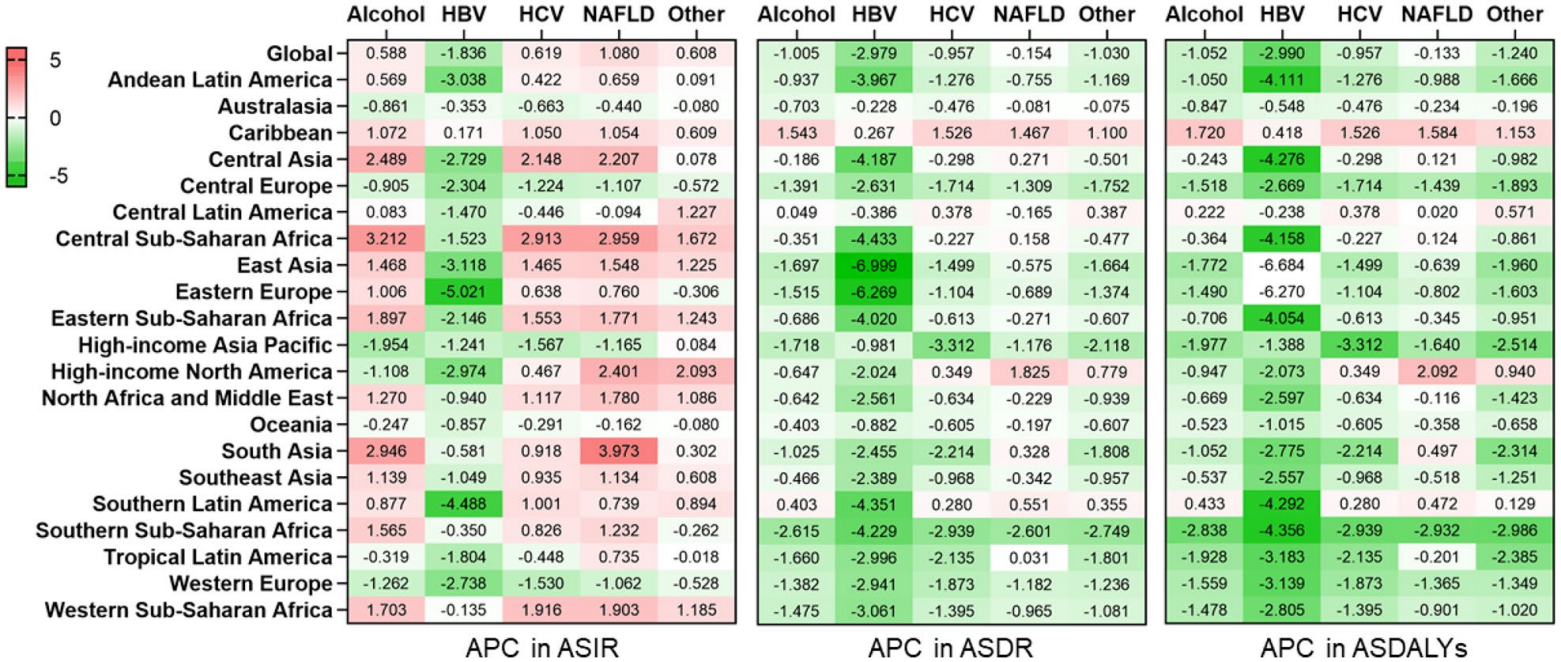
Annual percentage change (APC) in ASIR, ASDR and ASDALYs for cirrhosis due to different causes from 2010 through 2019. Box filled with red (APC > 0) indicates a worsening trend, while box filled with green (APC < 0) indicates an improving trend.

Temporal trends in age-standardized rates regarding the incidence, death, and DALYs of cirrhosis grouped by cause are depicted in Figure 4. During this period, the global ASIR for HBV-related cirrhosis significantly decreased (APC: −1.836 [95%CI −2.239 to −1.432]), whereas the ASIR for NAFLD increased significantly (APC:1.080 [95%CI 0.913–1.249]) over the study period (Supplementary Table 2). The largest decrease in the ASIR of HBV-induced cirrhosis was observed in Eastern Europe (APC −5.021 [95%CI −5.597 to −4.441]), Southern Latin America (APC −4.488 [95%CI −5.236 to −3.734]), and East Asia (APC −3.118 [95% CI −3.794 to −2.438]) (Figure 6, Appendix 4). South Asia (APC 3.973 [95%CI 3.236–4.715]), Central Sub-Saharan Africa (APC 2.959 [95%CI 2.165–3.760]), and Central Asia (APC 2.207 [95%CI 2.074–2.341]) exhibited the highest increase in ASIR of NAFLD-related cirrhosis. ASIRs for HCV- and alcohol-related cirrhosis also showed an increasing trend worldwide (APC for HCV 0.619 [95%CI 0.589–0.649], alcohol 0.588 [95%CI 0.187–0.990]) (Figure6, Appendix 4). Specifically, the incidence of cirrhosis due to alcohol use significantly increased in Central Sub-Saharan Africa (APC:3.212 [95% CI 2.331 to 4.101]), South Asia (APC:2.946 [95% CI: 0.681–5.261]), and Central Asia (APC:2.489 [95% CI 2.266 to 2.711]) (Figure 6, Appendix 4). The global ASDR for cirrhosis due to HBV (APC: −2.979 [95% CI −3.450 to −2.507]), alcohol (APC: −1.005 [95% CI −1.147 to −0.864]), HCV (APC: −0.957 [95% CI −1.110 to −0.805]), NAFLD (APC: −0.154 [95% CI −0.191 to −0.117]), and other aetiologies (APC: −1.030 [95% CI −1.123 to −0.937]) generally decreased (Supplementary Table 4). Interestingly, the ASDR of cirrhosis due to any cause increased in the Caribbean population (Figure 6, Appendix 5). High-income North America also exhibited a significant increase in the ASDR of NAFLD-associated cirrhosis (APC 1.825 [95%CI 1.462 to 2.189]) (Figure 6, Appendix 5). The patterns of ASDALYs of cirrhosis due to different causes were similar to those of ASDR by aetiology at the global and regional levels (Figure 6, Appendix 6).

## Discussion

Liver disease is responsible for around two million deaths annually across the globe. Out of these, one million deaths are caused by complications of cirrhosis, while the other million are attributed to viral hepatitis and hepatocellular carcinoma [4]. Nevertheless, it is important to note that cirrhosis, a major contributor to global health loss, is a largely preventable and often overlooked condition. Unlike previous studies focusing on specific regions, countries or causes of liver cirrhosis, the current study offers a comprehensive worldwide perspective on the burden of cirrhosis and the influence of different aetiologies over a 10-year period from 2010 to 2019.

Globally, in 2019, there were approximately 2.05 million incident cases, 1.47 million deaths and 46.19 million DALYs. The global age-standardized rates for incidence, mortality, and DALYs were 25.35, 18.00, and 56.43 per 100,000 population, respectively. Compared to the cirrhosis burden in 2010, there was a 12.9% increase in the absolute number of new cirrhosis cases and a 9.7% increase in cirrhosis-related deaths, although a decreased age-standardized death rate was observed during this period. It is highly conceivable that the growth and ageing of the world population may have contributed to the observed disparities in the trends between absolute counts and age-standardized rates.

In addition to the global perspective, we assessed the burden of cirrhosis at regional and national levels. Central Asia experienced the highest rates for both the incidence and mortality of cirrhosis at the regional level, attributed to the increasing trend in alcohol consumption that has accompanied economic development in many Asian countries (such as China and India) where alcohol control regulations are weak. Additionally, certain Asian populations with genetic polymorphisms in *ALDH1* and *ADH1B* have a higher risk of cirrhosis, further contributing to the burden of cirrhosis in the Asia-Pacific region [16,17]. Our results also revealed that the incidence rate of alcohol-related cirrhosis continuously increased in different areas in Asia (Central Asia, South Asia, and East Asia) between 2010 and 2019. These results emphasize the need to address the issue of stringent alcohol regulation to mitigate the worsening cirrhosis problem caused by alcohol consumption in certain Asian countries. A population-based study focusing on the burden of liver disease across 35 European countries reported an increasing incidence of cirrhosis [18]. In contrast, our study indicated that the incidence of cirrhosis and death generally decreased between 2010 and 2019 in Eastern Europe.

Analysis of disparities in cirrhosis burden according to gender revealed that the overall incidence, death, DALYs, and their age-standardized rates were higher in males than females. This difference can be explained by hormonal factors and a lower prevalence of high-risk lifestyles (such as smoking and alcohol abuse) among females. Our results also corroborated that the highest burden of cirrhosis among males and females occurred in the middle-aged population and that the age-standardized death rate increased steadily with increased age. This emphasizes the need for more policies focusing on these specific age groups.

It is now understood that the regional development level is also an important factor associated with cirrhosis burden. A negative correlation between mortality rate and SDIs was observed in the present study, although the incidence was not related to SDIs. Healthcare accessibility, health infrastructure, and educational level may account for the higher burden of cirrhosis-related deaths and DALY in lower SDI regions. A recent study using the GBD 2019 data also reported a more favourable quality-of-care index (QCI) was favourable in higher SDI countries (QCI = 86.8) compared with lower SDI countries (QCI = 60.1) [19]. Meanwhile, for the underlying causes of cirrhosis, the highest QCI was associated with alcohol use, followed by HBV and NAFLD, with QCIs of 86.1, 85.3, and 81.1, respectively [19]. These data suggest that countries, mainly in developing regions, should organize actions to control the burden of cirrhosis and its underlying causes and improve their quality of life.

Besides, we assessed the effects of different aetiologies on cirrhosis from a global perspective. Current evidence suggests that alcohol-associated cirrhosis has been the major cause of liver cirrhosis worldwide over the last 10 years. According to World Health Organization data, the worldwide average consumption of alcohol was 32.8 g per person per day in 2018 [20]. Among individuals consuming harmful amounts of alcohol in 2020, 59·1% (54·3–65·4) were aged 15–39 years [21]. Therefore, the prevalence of alcohol-related cirrhosis is expected to persist in the coming years. To mitigate this burden, it is recommended to implement measures such as raising the prices of alcoholic beverages or imposing higher taxes on them. Additionally, promoting education on the hazards of alcohol consumption habit is essential in reducing the impact of alcohol-related cirrhosis. Based on our data, HBV and HCV account for almost half of the global burden of cirrhosis. A recent systematic review and meta-analysis based on publications reporting 1376503 patients with cirrhosis from 86 countries or territories revealed that many cases had HBV (42%) and HCV (21%) infections [22]. However, there has been a notable positive trend worldwide in reducing the incidence and mortality rates associated with HBV infection, primarily due to widespread vaccination coverage and the effectiveness of antiviral therapies. Additionally, advancements in nucleic acid testing and the availability of highly effective oral antiviral medications against HCV have the potential to further reduce the burden of HCV-related cirrhosis. Besides, the prevalence of obesity and diabetes has increased worldwide [7,8]. It is widely believed that NAFLD will emerge as a leading cause of cirrhosis in the future. Consistently, NAFLD exhibited the most significant increase in cirrhosis-related death (+25.1%) and DALYs (+21.0%) over the study period. However, it was still the least common cause of incident cirrhosis (6.6%) and cirrhosis-related death (9.1%). Similarly, a recent systematic review and meta-analysis based on population studies suggested a globally increased NAFLD incidence in recent years [23]. Indeed, besides cirrhosis, NAFLD has been established as an important cause of liver cancer worldwide [24]. Therefore, urgent measures are required globally to tackle modifiable risk factors and reduce the growing burden of NAFLD-related cirrhosis. For other causes of cirrhosis, including autoimmune liver diseases, the incidence rate slightly increased (APC:0.608 [95% 0.581 to 0.635]) and death rates (APC: −1.030 [95% −1.123 to − 0.937]) decreased globally. Due to the classification of autoimmune liver diseases under the combined category of “other causes” in the GBD database, a specific analysis of the global burden of cirrhosis caused by autoimmune liver diseases was not feasible. To date, data on the global epidemiology of autoimmune liver disease are mainly available at regional or national levels. In Northern Europe, the point prevalence of autoimmune hepatitis (AIH) was estimated to be 10-–20 per 100,000 individuals and the incidence at 1–2 per 100000 person-years [25,26]. A recent study using the Optum cohort reported that the age- and sex-standardized prevalence of AIH was 26.6 per 100,000 persons with an incidence of 4.0 per 100,000 person-years from 2008 to 2018 in the USA [27]. A recent meta-analysis reported that the pooled point prevalence rate of primary biliary cholangitis in European countries was 22.27 (95% CI 17.98–27.01) cases per 100,000 inhabitants, and the pooled annual incidence rate was 1.87 (95% CI 1.46–2.34) new cases per 100,000 inhabitants [28]. Regarding the epidemiology of primary sclerosing cholangitis (PSC), Palak JT and colleagues reported that the epidemiology of PSC varies globally, but a rising trend in the incidence and prevalence was observed across Europe and North America [29].

Our study has several limitations and shortcomings that should be acknowledged. First, this study relied on GBD estimates; therefore, the availability and quality of primary data used in the DisMod-MR 2.1 model may influence the accuracy and robustness of the GBD estimates. Accordingly, our results should be interpreted with caution. Second, cirrhosis risk factors (such as tobacco smoking, obesity, diabetes, and genetic or metabolic causes) were not included in the GBD database, and their impact on the cirrhosis burden could not be estimated. Third, in contrast to GBD 2017[9], GBD 2019 modelled compensated and decompensated cirrhosis together. Stratification of the clinical stage of cirrhosis is recommended to clarify the burden of cirrhosis because decompensated cirrhosis carries a higher economic burden and negatively impacts the quality of life.

## Conclusions

In summary, cirrhosis remains a major public health issue worldwide, although geographical and sociodemographic variations in the cirrhosis burden prevail. Overall, the frequency of new cases, deaths, and DALYs from liver cirrhosis increased substantially between 2010 and 2019, while age-standardized rates of incidence increased, but death and DALYs decreased at the global level. The incidence of NAFLD-induced cirrhosis is rapidly increasing, even though it currently represents the smallest proportion. This emerging trend highlights NAFLD as an important contributor to liver cirrhosis, and its effects are expected to become more pronounced in the coming decade.

## Supplementary Material

Supplemental Material

## Data Availability

The datasets generated during and/or analyzed during the current study are available from the corresponding author on reasonable request.

## Registry and the registration no. of the study/trial

N/A

## Research involving recombinant DNA

N/A

## Data transparence statement

All data supporting the findings of this study are available within the article and its supplementary information files or from the corresponding author on reasonable request.
