# Supplementary material for: Changing epidemiology of cirrhosis from 2010 to 2019: results from the Global Burden Disease study 2019
Source: Ann Med. 2023 Aug 30;55(2):2252326. doi: 10.1080/07853890.2023.2252326 (PMC10469427; doi:10.1080/07853890.2023.2252326)
Supplement: Supplemental Material [file IANN_A_2252326_SM9767.zip › Supplementary Material.docx]

**Supplementary Materials**

**Supplementary Table**

Supplementary Table 1. Global and regional burden of cirrhosis incidence for both sexes and changes in incident cases and age-standardized incidence rate from 2010 to 2019

Supplementary Table 2. Global burden of cirrhosis incidence and changes in incident number and age-standardized incidence rate from 2010 to 2019 by sex, etiology and socio-demographic index

Supplementary Table 3. Global and regional burden of cirrhosis-related death for both sexes and changes in death number and age-standardized death rate from 2010 to 2019

Supplementary Table 4. Global burden of cirrhosis-related death and changes in death number and age-standardized death rate from 2010 to 2019 by sex, etiology and socio-demographic index

Supplementary Table 5. Global and reginal burden of disability adjusted life years (DALYs) of cirrhosis for both sexes and changes in DALYs and age-standardized DALYS from 2010 to 2019

Supplementary Table 6. Global burden of disability adjusted life years (DALYs) of cirrhosis and changes in DALYs and age-standardized DALYs rate from 2010 to 2019 by sex, etiology and socio-demographic index

**Appendixes**

Appendix 1. Burden of cirrhosis incidence for both sexes and changes in incident cases and age-standardized incidence rate from 2010 to 2019 across 204 countries. (.xlsx file)

Appendix 2. Burden of cirrhosis-related death for both sexes and changes in death number and age-standardized death rate from 2010 to 2019 across 204 countries. (.xlsx file)

Appendix 3. Burden of disability adjusted life years (DALYs) of cirrhosis for both sexes and changes in DALYs and age-standardized DALYS from 2010 to 2019 across 204 countries. (.xlsx file)

Appendix 4. Source data of the annual percentage change in age-standardized incidence rate for cirrhosis due to different causes from 2010 through 2019, at global and regional levels. (.xlsx file)

Appendix 5. Source data of the annual percentage change in age-standardized death rate for cirrhosis due to different causes from 2010 through 2019, at global and regional levels. (.xlsx file)

Appendix 6. Source data of the annual percentage change in age-standardized DALYs rate for cirrhosis due to different causes from 2010 through 2019, at global and regional levels. (.xlsx file)

**Supplementary Table 1. Global and regional burden of cirrhosis incidence for both sexes and changes in incident cases and age-standardized incidence rate (ASIR) from 2010 to 2019**

| **Location** | **2010** | | **2019** | | **Percentage changes in the number** | **Annual percentage change in ASIR (95% CI)** |
| --- | --- | --- | --- | --- | --- | --- |
|  | **No. incident cases (95% UI)** | **ASIR per 100000 population (95% UI)** | **No. incident cases (95% UI)** | **ASIR per 100000 population (95% UI)** |  |  |
| **Global** | 1816475 (1525479 to2133630) | 25.19 (21.21 to 29.53) | 2051554 (1661430 to 2478127) | 25.35 (20.78 to 30.44) | 12.94 | 0.104 (0.001 to 0.204) |
| **21 GBD Regions** |  |  |  |  |  |  |
| Andean Latin America | 15503 (14040 to 17052) | 31.76 (28.66 to 35.07) | 19973 (17615 to 22665) | 32.25 (28.4 to 36.69) | 28.83 | 0.156 (0.083 to 0.230) |
| Australasia | 3211 (2818 to 3609) | 10.69 (9.48 to 11.87) | 3315 (2795 to 3818) | 10.26 (8.75 to 11.72) | 3.24 | -0.587 (-0.979 to -0.193) |
| Caribbean | 9246 (8120 to 10384) | 20.26 (17.88 to 22.69) | 10843 (9202 to 12553) | 21.85 (18.56 to 25.08) | 17.27 | 0.896 (0.807 to 0.985) |
| Central Asia | 45934 (40654 to 51199) | 54.08 (48.45 to 59.47) | 58160 (51498 to 64782) | 59.06 (52.3 to 66.01) | 26.62 | 0.946 (0.742 to 1.151) |
| Central Europe | 41333 (37389 to 45092) | 32.35 (29.18 to 35.38) | 37556 (33208 to 41705) | 29.11 (25.85 to 32.45) | -9.14 | -1.175 (-1.283 to -1.066) |
| Central Latin America | 91019 (77140 to 105516) | 40.98 (34.72 to 47.69) | 106174 (85022 to 127868) | 40.76 (32.74 to 48.98) | 16.65 | 0.026 (-0.058 to 0.006) |
| Central Sub-Saharan Africa | 21000 (17737 to 24467) | 23.81 (19.07 to 28.83) | 30834 (25653 to 36784) | 26.97 (21.49 to 33.11) | 46.83 | 1.470 (1.346 to 1.595) |
| East Asia | 417149 (324932 to 512746) | 22.88 (18.19 to 27.86) | 424356 (321444 to 529849) | 22.51 (17.71 to 27.56) | 1.73 | -0.125 (-0.500 to 0.252) |
| Eastern Europe | 69953 (53772 to 89582) | 32.12 (24.77 to 40.87) | 66873 (47654 to 90005) | 31.27 (23.38 to 41.10) | -4.40 | -0.330 (-0.480 to -0.180) |
| Eastern Sub-Saharan Africa | 61405 (50041 to 73758) | 25.51 (19.36 to 32.66) | 86022 (67756 to 105518) | 27.15 (19.8 to 35.41) | 40.09 | 0.745 (0.676 to 0.814) |
| High-income Asia Pacific | 57920 (49992 to 65740) | 28.23 (24.59 to 31.74) | 50826 (41597 to 60070) | 25.15 (21.29 to 29.18) | -12.25 | -1.278 (-1.322 to -1.234) |
| High-income North America | 96099 (82284 to 110628) | 24.76 (21.84 to 28) | 98637 (81095 to 116830) | 25.62 (21.47 to 30.08) | 2.64 | 0.462 (0.129 to 0.795) |
| North Africa and Middle East | 114656 (98817 to 132290) | 26.69 (22.3 to 31.52) | 160140 (133538 to 190700) | 28.68 (23.58 to 34.88) | 39.67 | 0.792 (0.773 to 0.811) |
| Oceania | 927 (784 to 1076) | 8.79 (7.39 to 10.28) | 1124 (935 to 1324) | 8.5 (7.05 to 10.00) | 21.25 | -0.383 (-0.427 to -0.340) |
| South Asia | 337294 (253949 to 429329) | 21.63 (15.93 to 27.79) | 413984 (299626 to 539338) | 22.98 (16.68 to 29.92) | 22.74 | 0.717 (0.527 to 0.907) |
| Southeast Asia | 152315 (122484 to 182348) | 23.87 (19.02 to 28.64) | 181539 (142578 to 219744) | 24.76 (19.5 to 30.00) | 19.19 | 0.415 (0.381 to 0.449) |
| Southern Latin America | 19084 (17269 to 20830) | 29.54 (26.73 to 32.19) | 22198 (19634 to 24757) | 30.5 (27.07 to 34.08) | 16.32 | 0.337 (0.311 to 0.363) |
| Southern Sub-Saharan Africa | 10460 (8126 to 12928) | 15.11 (11.42 to 19.16) | 12183 (9242 to 15403) | 15.57 (11.7 to 19.87) | 16.47 | 0.361 (0.247 to 0.476) |
| Tropical Latin America | 45124 (34977 to 55923) | 20.63 (16.03 to 25.59) | 50187 (36235 to 65545) | 19.79 (14.41 to 25.57) | 11.22 | -0.433 (-0.478 to -0.389) |
| Western Europe | 134567 (123643 to 145797) | 27.47 (25.37 to 29.49) | 116178 (102873 to 129246) | 24.45 (21.91 to 26.93) | -13.67 | -1.242 (-1.294 to -1.190) |
| Western Sub-Saharan Africa | 72277 (60117 to 86090) | 24.31 (19.16 to 30.28) | 100453 (80813 to 121906) | 26.22 (19.92 to 33.23) | 38.98 | 0.902 (0.791 to 1.014) |

No. number, UI uncertainty interval, CI confidence interval

**Supplementary Table 2. Global burden of cirrhosis incidence and changes in incident number and age-standardized incidence rate (ASIR) from 2010 to 2019 by sex, etiologies and socio-demographic index**

|  | **2010** | | **2019** | | **Changes in the number (% with 95% CI)** | **Annual percentage change in ASIR (95% CI)** |
| --- | --- | --- | --- | --- | --- | --- |
|  | **No. incident cases (95% UI)** | **ASIR per 100000 population (95% UI)** | **No. incident cases (95% UI)** | **ASIR per 100000 population (95% UI)** |  |  |
| **Sex** | | | | | | |
| Male | 1089640 (898553 to 1297727) | 29.81 (24.77 to 35.32) | 1206125 (964223 to 1464589) | 29.67 (23.86 to 35.98) | 10.7 | -0.042 (-0.192 to 0.109) |
| Female | 726835 (615149 to 848432) | 20.93 (17.34 to 23.93) | 845429 (687514 to 1016947) | 20.14 (17.22 to 25.15) | 16.3 | 0.259 (0.177 to 0.340) |
| **Etiology** | | | | | | |
| Alcohol | 371376 (280943 to 472399) | 5.09 (3.88 to 6.47) | 436056 (314529 to 579116) | 5.24 (3.78 to 6.94) | 17.4 | 0.588 (0.187 to 0.990) |
| Hepatitis B | 420313 (315689 to 535854) | 5.78 (4.34 to 7.33) | 405891 (285248 to 536699) | 4.91 (3.46 to 6.47) | -0.34 | -1.836 (-2.239 to -1.432) |
| Hepatitis C | 459396 (353853 to 575992) | 6.31 (4.88 to 7.91) | 551689 (409296 to 711000) | 6.67 (4.98 to 8.56) | 20.1 | 0.619 (0.589 to 0.649) |
| NAFLD | 107308 (71226.3 to 161807) | 1.48 (0.99 to 2.22) | 135991 (88574 to 206295) | 1.63 (1.06 to 2.45) | 26.7 | 1.080 (0.913 to 1.249) |
| Other causes | 458083 (365163 to 561361) | 6.53 (5.26 to 8.03) | 521927 (407225 to 655285) | 6.90 (5.44 to 8.55) | 13.9 | 0.608 (0.581 to 0.635) |
| **Socio -demographic index (SDI)** | | | | | | |
| Low SDI | 173263 (141927 to 208071) | 22.53 (17.66 to 27.95) | 233081 (186145 to 284261) | 24.07 (18.49 to 30.18) | 34.5 | 0.782 (0.676 to 0.888) |
| Low to middle SDI | 358914 (288707 to 435142) | 23.99 (19.16 to 29.42) | 439460 (343041 to 544076) | 25.30 (19.68 to 31.38) | 22.4 | 0.618 (0.496 to 0.739) |
| Middle SDI | 598629 (484162 to 716820) | 25.40 (20.70 to 30.50) | 688560 (544115 to 840878) | 25.76 (20.56 to 31.34) | 15.0 | 0.181 ( to 0.007 to 0.369) |
| High to middle SDI | 407158 (340632 to 477361) | 25.61 (21.79 to 29.82)) | 421105 (340308 to 507295) | 24.90 (20.52 to 29.90) | 3.4 | -0.323 (-0.451 to -0.194) |
| High SDI | 277658 (249011 to 306139) | 25.41 (22.96 to 27.91) | 268353 (231077 to 305723) | 23.91 (20.82 to 27.14) | -3.4 | -0.657 ( -0.737 to -0.576) |

No. number, UI uncertain interval, CI confidence interval, NAFLD non-alcoholic fatty liver disease

**Supplementary Table 3. Global and regional burden of cirrhosis-related death for both sexes and changes in death number and age-standardized death rate (ASDR) from 2010 to 2019**

| **Location** | **2010** | | **2019** | | **Percentage changes in the number** | **Annual percentage change in ASDR (95% CI)** |
| --- | --- | --- | --- | --- | --- | --- |
|  | **No. death (95% UI)** | **ASDR per 100000 population (95% UI)** | **No. death (95% UI)** | **ASDR per 100000 population (95% UI)** |  |  |
| **Global** | 1341426 (1287440 to1405892) | 20.37 (19.45 to 21.36) | 1472012 (1374609 to 1578732) | 18 (16.8 to 19.3) | 9.73 | -1.392 ( -1.442 to -1.343) |
| **21 GBD Regions** |  |  |  |  |  |  |
| Andean Latin America | 11676 (10257 to 13104) | 27.96 (24.55 to 31.38) | 14052 (11229 to 17328) | 25.1 (20.1 to 30.9) | 20.35 | -1.170 ( -1.253 to -1.087) |
| Australasia | 2078 (1942 to 2196) | 5.61 (5.27 to 5.91) | 2512 (2298 to 2731) | 5.5 (5 to 5.9) | 20.89 | -0.368 ( -0.942 to 0.201) |
| Caribbean | 7117 (6051 to 8229) | 16.64 (14.17 to 19.21) | 9546 (7777 to 11409) | 18.5 (15 to 22.2) | 34.13 | 1.277 (1.067 to 1.488) |
| Central Asia | 29898 (28959 to 30795) | 46.69 (44.92 to 48.30) | 33910 (30488 to 37737) | 42.9 (38.5 to 47.5) | 13.42 | -0.939 ( -1.100 to -0.779) |
| Central Europe | 36414 (35554 to 37254) | 20.73 (20.27 to 21.21) | 33596 (29317 to 37900) | 17.7 (15.4 to 20) | to7.74 | -1.805 ( -2.260 to -1.349) |
| Central Latin America | 51143 (48906 to 53232) | 28.3 (26.97 to 29.52) | 68062 (58609 to 78330) | 28.3 (24.5 to 32.6) | 33.08 | -0.072 ( -0.579 to 0.439) |
| Central Sub-Saharan Africa | 19011 (15005 to 23357) | 42.29 (33.21 to 51.58) | 22758 (17088 to 29114) | 37 (28 to 47.4) | 19.71 | -1.471 ( -2.000 to -0.938) |
| East Asia | 173405 (158291 to 190449) | 11.04 (10.06 to 12.08) | 164720 (140148 to 191748) | 8.2 (7 to 9.5) | to5.01 | -3.288 ( -3.483 to -3.093) |
| Eastern Europe | 83044 (81856 to 84164) | 29.14 (28.75 to 29.53) | 72718 (64995 to 81006) | 24.3 (21.7 to 27) | to12.43 | -2.218 ( -3.445 to -0.975) |
| Eastern Sub-Saharan Africa | 64591 (57947 to 74849) | 49.6 (44.29 to 57.4) | 76980 (66309 to 91419) | 44.1 (38.5 to 51.9) | 19.18 | -1.227 ( -1.373 to -1.081) |
| High-income Asia Pacific | 35320 (31755 to 38644) | 10.42 (9.58 to 11.22) | 36888 (32213 to 41082) | 8.7 (7.9 to 9.4) | 4.44 | -2.075 ( -2.306 to -1.843) |
| High-income North America | 59916 (57555 to 61825) | 12.28 (11.87 to 12.65) | 72738 (69313 to 75465) | 12.7 (12.2 to 13.1) | 21.4 | 0.267 (0.172 to 0.362) |
| North Africa and Middle East | 90331 (78326 to 99459) | 30.74 (26.95 to 33.81) | 109707 (81390 to 135204) | 27.7 (21.1 to 33.9) | 21.45 | -1.140 ( -1.304 to -0.977) |
| Oceania | 924 (750 to 1128) | 13.88 (11.42 to 16.79) | 1153 (905 to 1446) | 13.2 (10.6 to 16.4) | 24.78 | -0.621 ( -0.712 to -0.531) |
| South Asia | 310247 (284855 to 335850) | 27.15 (24.77 to 29.45) | 348393 (306925 to 404846) | 23.5 (20.7 to 27.1) | 12.3 | -1.671 ( -1.848 to -1.493) |
| Southeast Asia | 161510 (150551 to 174446) | 33.36 (31.21 to 35.85) | 186154 (165362 to 207697) | 30.2 (26.9 to 33.5) | 15.26 | -1.166 ( -1.314 to -1.018) |
| Southern Latin America | 11933 (11421 to 12446) | 17.6 (16.86 to 18.33) | 14154 (13221 to 15117) | 17.3 (16.2 to 18.5) | 18.61 | -0.023 ( -0.208 to 0.162) |
| Southern Sub-Saharan Africa | 9847 (8872 to10929) | 20.35 (18.43 to 22.45) | 9231 (8230 to 10320) | 15.4 (13.8 to 17.2) | -6.26 | -3.031 ( -3.322 to -2.738) |
| Tropical Latin America | 34563 (33255 to 36210) | 18.14 (17.33 to 19.07) | 38778 (36546 to 41258) | 15.7 (14.8 to 16.8) | 12.2 | -1.725 ( -1.921 to -1.529) |
| Western Europe | 77157 (73031 to 81271) | 10.79 (10.32 to 11.28) | 77226 (72045 to 82464) | 9.4 (8.9 to 10) | 0.09 | -1.511 ( -1.727 to -1.294) |
| Western Sub-Saharan Africa | 71302 (57222 to 87437) | 45.34 (36.73 to 55.46) | 78737 (61695 to 99766) | 37.5 (30.3 to 46.5) | 10.43 | -2.183 ( -2.368 to -1.996) |

No. number, UI uncertainty interval, CI confidence interval

**Supplementary Table 4. Global burden of cirrhosis-related death and changes in death number and age-standardized death rate (ASDR) from 2010 to 2019 by sex, etiologies and socio-demographic index**

|  | **2010** | | **2019** | | **Changes in the Number (%)** | **Annual percentage change of ASDR (95% CI)** |
| --- | --- | --- | --- | --- | --- | --- |
|  | **No. Deaths (95% UI)** | **ASDR per 100000 population (95% UI)** | **No. Deaths (95% UI)** | **ASDR per 100000 population (95% UI)** |  |  |
| **Sex** | | | | | | |
| Male | 891589 (852584 to 934280) | 28.20 (26.90 to 29.50) | 969068 (899213 to 1045344) | 24.81 (23.07 to 26.75) | 8.7% | -1.452 (-1.509 to -1.396) |
| Female | 449837 (423289 to 479069) | 13.13 (12.33 to 13.99) | 502944 (459202 to 550914) | 11.69 (10.68 to 12.81) | 11.8% | -1.277 (-1.336 to -1.218) |
| **Etiology** | | | | | | |
| Alcohol | 325437 (278747 to 376049) | 4.92 (4.21 to 5.67) | 371964 (314703 to 438425) | 4.48 (3.81 to 5.28) | 14.3 | -1.005 ( -1.147 to -0.864) |
| Hepatitis B | 350244 (306799 to 399427) | 5.24 (4.61 to 6.00) | 331267 (278543 to 392127) | 4.03 (3.39 to 4.76) | -5.4 | -2.979 ( -3.450 to - 2.507) |
| Hepatitis C | 342642 (298988 to 392747) | 5.20 (4.54 to 5.96) | 395022 (335832 to 458584) | 4.82 (4.09 to 5.57) | 15.3 | -0.957 ( -1.110 to - 0.805) |
| NAFLD | 107312 (77816 to 142370) | 1.68 (1.23 to 2.22) | 134241 (96483 to 176920) | 1.66 (1.20 to 2.17) | 25.1 | -0.154 (- 0.191 to - 0.117) |
| Other causes | 215791 (173891 to 264615) | 3.32 (2.65 to 4.10) | 239518 (187991 to 302874) | 3.02 (2.38 to 3.78) | 11.0 | -1.030 ( -1.123 to - 0.937) |
| **Socio to demographic index (SDI)** | | | | | | |
| Low SDI | 165759 (149513 to 183169) | 38.14 (34.27 to 42.16) | 187884 (163817 to 215152) | 32.78 (28.90 to 37.11) | 13.4 | -1.635 ( -1.792 to - 1.478) |
| Low-middle SDI | 328957 (306979 to 351404) | 29.24 （27.21 to 31.33） | 376211 (342150 to 416612) | 26.21 (23.86 to 28.99) | 14.4 | -1.234 (-1.396 to -1.071) |
| Middle SDI | 417576 （397856 to 439686） | 22.24 (21.04 to 23.35) | 469614 (427787 to 516789) | 19.23 (17.45 to 21.15) | 12.5 | -1.636 (-1.721 to - 1.550) |
| High-middle SDI | 260178 (252189 to 268452)) | 15.99 (15.46 to 16.53) | 251935 (236058 to 269275) | 12.81 (12.00 to 13.68) | -3.2 | -2.423 ( -2.837 to - 2.007) |
| High SDI | 168236 (159625 to 176451) | 11.60 (11.10 to 12.09) | 185503 (173741 to 196100) | 10.77 (10.23 to 11.31) | 10.3 | -0.835 (-0.919 to -0.751) |

No. number, NAFLD non to alcoholic fatty liver disease, UI uncertain interval, CI confidence interval

**Supplementary Table 5. Global and reginal burden of disability adjusted life years (DALYs) of cirrhosis for both sexes and changes in DALYs and age-standardized DALYs rate (ASDALYs) from 2010 to 2019**

| **Location** | **2010** | | **2019** | | **Percentage changes in the number** | **Annual percentage change in ASDR (95% CI)** |
| --- | --- | --- | --- | --- | --- | --- |
|  | **No. DALYs (95% UI)** | **ASDALYs per 100000 population (95% UI)** | **No. DALYs (95% UI)** | **ASDALYs per 100000 population (95% UI)** |  |  |
| **Global** | 44110812 (42448751 to 46337063) | 639.86 (615.71 to 671.51) | 46189416 (43027109 to 49551292) | 560.4 (521.9 to 602) | 4.71 | -1.498 ( -1.557 to -1.439) |
| **21 GBD Regions** |  |  |  |  |  |  |
| Andean Latin America | 324257 (284968 to 364355) | 727.35 (639.50 to 817.76) | 373058 (294137 to 465085) | 639.1 (506.1 to 795.5) | 15.05 | -1.426 ( -1.621 to -1.231) |
| Australasia | 55512 (52490 to 58185) | 159.68 (151.31 to 167.32) | 63253 (58694 to 67742) | 153.4 (142.8 to 164.1) | 13.94 | -0.539 ( -0.860 to -0.217) |
| Caribbean | 211881 (174306 to 251163) | 486.37 (399.90 to 576.85) | 278278 (219587 to 341080) | 545.7 (429.1 to 672.6) | 31.34 | 1.355 (1.221 to 1.488) |
| Central Asia | 1057389 (1028540 to 1086216) | 1458.21 (1415.26 to 1498.93) | 1173145 (1054258 to 1309149) | 1318.2 (1187 to 1467.1) | 10.95 | -1.262 ( -1.325 to -1.200) |
| Central Europe | 1090455 (1070641 to 1113652) | 655.55 (644.02 to 669.56) | 964512 (839764 to 1091671) | 554.8 (483.1 to 627.7) | -11.55 | -1.957 ( -2.436 to -1.475) |
| Central Latin America | 1575377 (1525809 to 1633168) | 806.8 (779.22 to 836.92) | 2031944 (1740913 to 2343602) | 816.6 (700.8 to 940.4) | 28.98 | 0.089 ( -0.502 to 0.684) |
| Central Sub-Saharan Africa | 712845 (567826 to 878803) | 1246.75 (980.21 to 1533.07) | 843049 (629422 to 1106950) | 1089.7 (821 to 1392.7) | 18.27 | -1.454 ( -1.541 to -1.367) |
| East Asia | 5259889 (4797141 to 5815520) | 308.01 (281.81 to 339.18) | 4698614 (3982096 to 5513225) | 227.9 (193.8 to 266.2) | -10.67 | -3.301 (-3.550 to -3.052) |
| Eastern Europe | 3026099 (2988314 to 3072090) | 1109.4 (1095.96 to 1127.05) | 2575335 (2298785 to 2874695) | 919.5 (820.7 to 1026.8) | -14.9 | -2.330 (-3.612 to -1.032) |
| Eastern Sub-Saharan Africa | 2231419 (1997167 to 2600826) | 1343.53 (1205.56 to 1560.28) | 2608125 (2191259 to 3151159) | 1178.4 (1015.5 to 1402.5) | 16.88 | -1.422 (-1.799 to -1.044) |
| High-income Asia Pacific | 833512 (789137 to 879279) | 283.52 (271.7 to 297.81) | 776896 (717258 to 824271) | 230.20 (216.00 to 242.90) | -6.79 | -2.322 ( -2.479 to -2.165) |
| High to income North America | 1700058 (1656169 to 1737671) | 364.75 (355.39 to 372.30) | 1955333 (1892680 to 2014259) | 371.40 (360.20 to 381.80) | 15.02 | 0.181 (0.093 to 0.269) |
| North Africa and Middle East | 2452351 (2093752 to 2712626) | 689.54 (591.03 to 760.81) | 2878426 (2126424 to 3565004) | 616.8 (457.7 to 761.6) | 17.37 | -1.233 ( -1.414 to -1.052) |
| Oceania | 37528 (30220 to 46236) | 468.49 (380.27 to 571.68) | 45978 (35985 to 57692) | 440.8 (347.9 to 550.5) | 22.52 | -0.720 ( -0.805 to -0.635) |
| South Asia | 11806344 (10902341 to 12745694) | 884.8 (815.48 to 955.12) | 12436281 (10954317 to 14414136) | 750.6 (662.2 to 867.9) | 5.34 | -1.881 ( -2.495 to -1.263) |
| Southeast Asia | 5428705 (5031313 to 5930875) | 975.16 (904.32 to 1062.69) | 5915349 (5207219 to 6642923) | 867 (765.1 to 970.7) | 8.96 | -1.288 ( -1.442 to -1.133) |
| Southern Latin America | 321572 (311451 to 333130) | 483.77 (468.63 to 500.99) | 372368 (351788 to 394659) | 471.6 (446.4 to 499.4) | 15.8 | -0.160 ( -0.317 to -0.002) |
| Southern Sub- Saharan Africa | 343642 (304850 to 386075) | 619.18 (557.5 to 689.94) | 310794 (272944 to 353505) | 458.7 (405.3 to 516.6) | -9.56 | -3.248 ( -3.554 to -2.941) |
| Tropical Latin America | 1131062 (1100349 to 1175793) | 560.45 (544.16 to 583.39) | 1193340 (1134408 to 1261300) | 473.9 (449.6 to 501.1) | 5.51 | -2.095 ( -2.360 to -1.830) |
| Western Europe | 1944542 (1880660 to 2019602) | 304.51 (295.93 to 315.39) | 1832992 (1752144 to 1930380) | 260.9 (250.6 to 273.6) | -5.74 | -1.708 ( -1.947 to -1.468) |
| Western Sub-Saharan Africa | 2566374 (2040860 to 3136343) | 1258.26 (1004.62 to 1549.86) | 2862345 (2196426 to 3688872) | 1052.2 (823.2 to 1337.6) | 11.53 | -2.024 ( -2.189 to -1.859) |

No. number, UI uncertainty interval, CI confidence interval

**Supplementary Table 6. Global burden of disability adjusted life years (DALYs) of cirrhosis and changes in DALYs and age-standardized DALYs rate from 2010 to 2019 by sex, etiologies and socio-demographic index**

|  | **2010** |  | **2019** |  | **Changes in the Number (%)** | **Annual percentage change of ASDALYs (95% CI)** |
| --- | --- | --- | --- | --- | --- | --- |
|  | **No. DALYs (95% UI)** | **ASDALYs per 100000 population (95% UI)** | **No. DALYs (95% UI)** | **ASDALYs per 100000 population (95% UI)** |  |  |
| **Sex** | | | | | | |
| Male | 30469446 (2914381 to 32020458) | 895.05 (856.24 to 940.39) | 31781079 (29366252 to 34438138) | 783.31 (723.85 to 849.07) | 4.3 | -1.527 ( -1.609 to -1.446) |
| Female | 13641367 (12881064 to 14436333) | 391.71 (369.85 to 415.55) | 14408336 (13159621 to 15759496) | 343.96 (313.74 to 376.74) | 5.6 | -1.424 ( -1.503 to -1.346) |
| **Etiology** | | | | | | |
| Alcohol | 10086282 (8600658 to 11705412) | 147.48 (125.85 to 170.48) | 11186601 (9447033 to 13125598) | 133.31 (112.68 to 156.17) | 11.9 | -1.052 (-1.179 to -0.925) |
| Hepatitis B | 11741212 (10229886 to 13322345) | 168.62 (146.87 to 191.28) | 10786755 (8991170 to 127156662) | 129.85 (108.27 to 152.95) | -8.1 | -2.99 (-3.493 to -2.486) |
| Hepatitis C | 11005349 (9594732 to 12578328) | 159.24 (138.91 to 182.14) | 12157161 (10325549 to 14166608) | 146.25 (124.42 to 169.83) | 10.5 | -0.957 (-1.11 to -0.805) |
| NAFLD | 2993284 (2132976 to 4000425) | 44.29 (31.83 to 59.01) | 3621472 (2585375 to 4862918) | 43.69 (31.28 to 58.38) | 21.0 | -0.133 (-0.222 to -0.044) |
| Other causes | 8284684 (7022654 to 9785600) | 44.292897 (31.83 to 59.01) | 8437427 (6936187 to 10236889) | 107.34 (88.77 to 129.65) | 1.8 | -1.24 (-1.359 to -1.121) |
| **Socio-demographic index (SDI)** | | | | | | |
| Low SDI | 6136438 (5535703 to 6800764) | 1117.53 (1008.26 to 1233.14) | 6773037 (5866959 to 7846211) | 949.51 (826.87 to 1088.95) | 10.4 | -1.752 ( -1.913 to -1.590) |
| Low-middle SDI | 11918798 (11124128 to 12721799) | 924.14 (863.35 to 986.00) | 12974479 (11742205 to 14369549) | 817.87 (739.83 to 907.80) | 8.9 | -1.367 ( -1.765 to -0.967) |
| Middle SDI | 13182620 (12618049 to 13934482) | 622.32 (595.32 to 655.25) | 14074007 (12898464 to 15406240) | 538.88 (494.31 to 589.66) | 6.8 | -1.608 (-1.710 to -1.505) |
| High-middle SDI | 8313508 (8103171 to 8557860) | 506.78 (493.95 to 522.16) | 7639449 （7168729 to 8166771） | 401.80 (377.56 to 429.53) | -8.1 | -2.599 (-3.164 to -2.030) |
| High SDI | 4536935 (4407725 to 4675228) | 337.92 (329.42 to 347.51) | 4702397 （4508829 to 4882152） | 307.80 (296.08 to 318.87) | 3.6 | -1.035 ( -1.183 to -0.887) |

No. number, NAFLD non to alcoholic fatty liver disease, UI uncertain interval, CI confidence interval
